# Editorial: Omics in plant-insect interactions

**DOI:** 10.3389/fpls.2024.1480678

**Published:** 2024-09-24

**Authors:** Shengli Jing, Guangcun He, Ming-shun Chen

**Affiliations:** ^1^ College of Life Sciences, Xinyang Normal University, Xinyang, China; ^2^ State Key Laboratory of Hybrid Rice, College of Life Sciences, Wuhan University, Wuhan, China; ^3^ Hard Winter Wheat Genetics Research Unit, USDA-ARS, Manhattan, KS, United States

**Keywords:** plant-insect interactions, multi-omics analysis, resistance mechanism, secondary metabolites, resistance genes

Insect herbivores and their host plants engage in a dynamic molecular conflict, wherein plants have evolved sophisticated defense mechanisms, while insects have developed strategies to suppress these defenses. These interactions involve intricate molecular processes that are a major focus of research, with the goal of improving pest control strategies.

Herbivorous insects, such as piercing-sucking hemipterans and chewing Lepidopterans, establish close associations with host plants to manipulate plant cellular processes for feeding and reproduction (Erb and Reymond, 2019). In response, plants have evolved complex immune systems composed of signaling pathways, resistance genes, and secondary metabolites. This ongoing molecular arms race between plants and insects is characterized by interactions among a diverse array of molecules from both organisms. Plant secondary metabolites and resistance genes serve as defense mechanisms against insect herbivory, while insects utilize detoxification enzymes and effectors to disrupt plant defenses, enhancing their adaptation.

This Research Topic explores the complex genetic, physical, metabolic, and molecular interactions between plants and insects, examined through advanced omics technologies such as genomics, transcriptomics, and metabolomics. The Research Topic includes 16 original research and review articles focusing on herbivorous insects (e.g., brown planthopper, aphids, psyllids) and their host plants (e.g., rice, maize, cotton), which are summarized below.

## Interactions between rice and brown planthopper

Eight articles in this Research Topic (one review and seven research articles) focus on the interaction between rice (*Oryza sativa* L.) and the brown planthopper (*Nilaparvata lugens* Stål, BPH). This interaction serves as a valuable model system for studying the molecular mechanisms underlying plant-insect interactions. The brown planthopper is a major pest that causes significant damage to rice crops (Cheng et al., 2013; Jing et al., 2017), and the deployment of resistant rice varieties has proven effective in managing this pest. To date, over 40 resistance loci and 17 resistance genes for brown planthopper have been identified and functionally characterized (Shar et al., 2023). The molecular mechanisms of these resistance genes represent a key area of research. This Research Topic includes articles on the molecular mechanisms involving four specific resistance genes: *Bph6*, *Bph14*, *Bph15*, and *Bph30*.

Four articles focus on *Bph15*, examining the specific cells and tissues involved in resistance, the role of *OsWRKY71* in *Bph15*-mediated resistance, the defense mechanisms against BPH populations with varying virulence levels, and transcriptomic analysis to elucidate the mechanisms behind *BPH14*/*BPH15*-conferred resistance. One article investigates how indole-3-acetic acid (IAA) negatively regulates *Bph30*-mediated resistance using combining transcriptomic and metabolomic analyses. Two additional articles (one review and one research) explore the impact of non-coding RNAs on the interaction between rice and brown planthopper, and one article identifies BPH resistance genes using Genome-wide association studies (GWAS) and evaluates the predictive ability of genomic selection for resistance to BPH.

Zha et al. used single-cell sequencing technology to analyze different cell types involved in BPH resistance, comparing the responses of leaf sheaths from susceptible (TN1) and resistant (YHY15) rice varieties 48 hours after infestation. The analysis identified 14,699 cells in TN1 and 16,237 cells in YHY15, which were grouped into nine cell-type clusters using cell-specific marker genes. Significant differences were observed between the two rice varieties in their resistance mechanisms to BPH. Further analysis revealed that different cell types employ distinct molecular mechanisms in response to BPH, enhancing the understanding of the molecular processes underlying rice resistance and accelerating the breeding of insect-resistant rice varieties.

Li et al. discovered that the transcription factor OsWRKY71 is highly responsive to BPH infestation, with early-induced expression in *Bph15* near-isogenic line (NIL) plants. OsWRKY71 localizes in the nucleus of rice protoplasts, and its knockout in the *Bph15*-NIL background using CRISPR-Cas9 technology resulted in compromised *Bph15*-mediated resistance. Transcriptome analysis indicated significant differences in the transcriptional response to BPH infestation between the *wrky71* mutant and the *Bph15*-NIL, affecting the expression of several defense-related genes. The study identified three potential participants (OsSTPS2, OsEXO70J1, and RGA2) in the BPH resistance pathway mediated by OsWRKY71 in *Bph15*-NIL plants, highlighting the crucial role of OsWRKY71 in *Bph15*-mediated resistance.

Yu et al. conducted miRNA and mRNA expression profiling to investigate the differential responses of YHY15 rice to both avirulent (biotype 1) and virulent (biotype Y) BPH. The study found that YHY15 rice exhibited a rapid response to biotype Y BPH infestation, with significant transcriptional changes within 6 hours. The biotype Y-responsive genes were enriched in photosynthetic processes, and biotype Y BPH infestation induced more intense transcriptional responses compared to biotype 1 BPH, affecting miRNA expression, defense-related metabolic pathways, phytohormone signaling, and multiple transcription factors. Callose deposition was also enhanced in biotype Y BPH-infested rice seedlings, indicating a heightened defense response. These findings provide comprehensive insights into the defense mechanisms of resistant rice against virulent BPH populations.

Hu et al. performed transcriptomic analysis to understand the mechanisms behind *Bph14*/*Bph15*-conferred resistance, using near-isogenic lines (NILs) of rice containing *Bph14* (B14), *Bph15* (B15), or both *Bph14* and *Bph15* (B1415), compared to their recurrent parent (RP) variety ‘Wushansimiao’. The study identified 14,492 differentially expressed genes (DEGs) across the profiles, with 531 DEGs common to the resistant NILs compared to the RP before and after BPH feeding. The DEGs related to BPH resistance were primarily enriched in defense response and oxidative stress pathways. Twenty-one DEGs were selected as candidate genes for BPH resistance based on their expression patterns and relevance from previous research. OsPOX8.1, one of the candidate genes, was validated in rice protoplasts, showing increased reactive oxygen species (ROS) accumulation, providing insights into the defense mechanisms activated by BPH resistance gene pyramiding in rice.

Shi et al. combined transcriptomic and metabolomic analyses to study *Bph30*-transgenic (Bph30T) rice plants and BPH-susceptible Nipponbare plants. Transcriptomic analysis revealed that many differentially expressed genes (DEGs) in susceptible rice plants were involved in plant hormone signal transduction, particularly in indole-3-acetic acid (IAA) signal transduction. Metabolomic analysis showed that differentially accumulated metabolites (DAMs) in the amino acids and derivatives category were down-regulated, while DAMs in the flavonoids category were up-regulated in resistant plants following BPH feeding. Combined analysis indicated that flavonoid and lignin biosynthesis are involved in resistance, while biosynthesis of various amino acids and IAA are involved in susceptibility. The study confirmed that exogenous IAA application weakened *Bph30*-mediated BPH resistance, demonstrating that IAA negatively regulates *Bph30*-mediated resistance through the shikimate and phenylpropanoid metabolism pathways.

Jing et al. summarized the roles of small RNAs in the rice-BPH interaction. Functional validation experiments indicated that these sRNAs fine-tune plant innate immunity by integrating R gene-mediated resistance, phytohormone signaling, callose deposition, reactive oxygen species (ROS) production, and secondary metabolite biosynthesis. Additionally, sRNAs are involved in key aspects of BPH biology, such as metamorphosis, wing polyphenism, molting, and reproductive development. The study also observed cross-kingdom RNAi in the rice-BPH interaction, suggesting that sRNAs ingested by BPH while feeding on rice may regulate BPH gene expression. These findings highlight the potential of HIGS and SIGS as promising agricultural pest control strategies.

Wu et al. focused on the regulatory roles of non-coding RNAs, specifically long non-coding RNAs (lncRNAs) and circular RNAs (circRNAs), in *Bph6*-transgenic (resistant, BPH6G) and Nipponbare (susceptible, NIP) rice plants infested by brown planthoppers. Genome-wide analysis identified 310 differentially expressed lncRNAs and 129 differentially expressed circRNAs between the resistant and susceptible rice plants. Dual-luciferase reporter assays revealed specific interactions between lncRNAs and microRNAs (miRNAs), such as lncRNA XLOC_042442 targeting miR1846c and lncRNA XLOC_028297 targeting miR530. The study predicted that 39 lncRNAs and 21 circRNAs could interact with 133 common miRNAs and compete for miRNA binding sites with 834 mRNAs, implicating these mRNAs in key biological processes such as cell wall organization, biogenesis, developmental growth, single-organism cellular processes, and stress responses. This study provides a comprehensive identification and characterization of lncRNAs and circRNAs in rice plants infested by BPH, laying a crucial foundation for future research on non-coding RNAs in the rice-BPH interaction.

Zhou et al. present a comprehensive resource on BPH resistance genes and offer practical recommendations for genomic selection in rice breeding programs. Through Genome-Wide Association Studies (GWAS), six loci were significantly associated with BPH resistance across three assessment criteria. Among these, two loci were novel, while others included previously identified BPH-resistant genes such as *Bph6*, *Bph32*, and *Bph37*. The accuracy of genomic prediction (GP) was influenced by the number of SNPs, training population size, and statistical models. To enhance the prediction of BPH resistance, it is recommended to increase SNP numbers beyond 26,000, expand the training population size beyond 737 individuals, and utilize random forest (RF) models. Optimizing these genomic selection strategies will facilitate the development of durable BPH-resistant rice varieties, thereby contributing to sustainable global rice production.

## Aphid-mediated tritrophic interactions

Two articles in this Research Topic examine aphids as vectors in tritrophic interactions. Zhao et al. investigate the effects of nitrogen fertilization on the emission of plant volatile organic compounds (VOCs) that mediate interactions among maize, aphids, and ladybirds. Specifically, 1-nonene was identified as a key compound attracting ladybirds, with its release positively correlated with the visitation rates of *Harmonia axyridis*. The study demonstrated that supplying 1-nonene to maize under low-nitrogen conditions increased the attractiveness of plants to ladybirds, highlighting the compound’s role in tritrophic interactions. The synthesis of 1-nonene is linked to salicylic acid (SA) and abscisic acid (ABA), indicating complex interactions within plant response mechanisms to nutrient availability and pest infestation. This research enhances our understanding of plant metabolic responses to nutrient levels and offers practical insights for improving pest management and crop production.

Pandey et al. explore the influence of host plants on the gene expression of the cotton aphid (*Aphis gossypii* Glover) when infected with the cotton leafroll dwarf virus (CLRDV). Using four host plants—cotton, hibiscus, okra, and prickly sida—the study revealed significant differences in gene expression among aphids acquiring the virus from different hosts. A total of 2,942 differentially expressed genes (DEGs) were identified, with varying DEG counts across hosts: 750 from cotton, 310 from hibiscus, 1,193 from okra, and 689 from prickly sida. Notably, more genes were overexpressed in aphids from cotton, hibiscus, and prickly sida, while more genes were underexpressed in aphids from okra. These findings underscore the complexity of vector-virus-host interactions and the pivotal role of host plants in influencing disease transmission.

## Psyllid-pathogen interactions

Two articles in this Research Topic focus on psyllids as vectors in vector-pathogen interactions. Li et al. examine the biochemical effects of *Candidatus Liberibacter* solanacearum (CLso) infection in potato psyllids (*Bactericera cockerelli*), vectors of diseases such as psyllid yellows, vein-greening (VG), and zebra chip (ZC). Using ultra-performance liquid chromatography tandem mass spectrometry (UPLC-MS/MS), the study identified 34 metabolites related to amino acid, carbohydrate, and lipid metabolism as potential biomarkers of CLso infection. Matrix-assisted Laser Desorption Ionization Mass Spectrometry Imaging (MALDI-MSI) mapped the spatial distribution of these biomarkers, revealing significant down-regulation of 15-keto-Prostaglandin E2 and alpha-D-Glucose in the abdomen of infected psyllids. These findings suggest mechanisms of immune suppression employed by CLso to evade detection and clearance by the psyllid’s immune system.

He et al. investigate the interactions between the Asian citrus psyllid (ACP) and *Candidatus Liberibacter* asiaticus (CLas), the causative agent of citrus greening disease. This research explores how different life stages of ACP influence gene expression in response to CLas infection. RNA sequencing (RNA-seq) revealed significant changes in gene expression, particularly in later nymphal stages (4-5) and teneral adults. A large number of genes related to defense mechanisms, developmental processes, and immune responses were found to be highly responsive to CLas infection. Understanding these gene expressions provides insights into how ACP manages the bacterial infection and contributes to disease transmission.

## Plant-herbivore interactions

Two articles in this Research Topic focus on interactions between herbivorous insects and their host plants. Liu et al. investigate how insect egg deposition affects plant defenses in willows (*Salix matsudana* ‘Zhuliu’). The study found that egg deposition by *Plagiodera versicolora* triggered plant defensive responses and enhanced the plant’s ability to cope with subsequent larval feeding. RNA-seq analysis revealed altered expression of genes related to stress responses and metabolic processes. Following larval feeding, increased activity of genes involved in phenylpropanoid biosynthesis and phytohormone signaling was observed, indicating that egg deposition primes the plant for heightened defense. Bioassays demonstrated that larvae feeding on leaves with prior egg deposition exhibited reduced performance, suggesting that the willow’s induced defenses effectively decrease larval survivability.

Sun et al. analyze the biochemical and molecular responses of daylilies (*Hemerocallis citrina* ‘Datong Huanghua’) to feeding by *Thrips palmi*. The study found significant reductions in soluble sugar, amino acid, and free fatty acid levels in daylily leaves after *T. palmi* feeding, alongside increases in secondary metabolites like tannins, flavonoids, and phenols. Key defense enzymes, including peroxidase (POD), phenylalanine ammonia lyase (PAL), and polyphenol oxidase (PPO), were significantly enhanced. RNA sequencing identified 1,894 differentially expressed genes (DEGs) associated with *T. palmi* feeding, with 698 predicted as transcription factors involved in stress responses. Weighted Gene Co-expression Network Analysis (WGCNA) highlighted 18 hub genes in key modules, potentially crucial for regulating defense responses.

## Review articles on plant-insect interactions

Two review articles summarize plant defenses and key insect proteins involved in interactions with host plants. Li et al. review current knowledge and advances in plant defenses against whiteflies, emphasizing the role of trichomes and acylsugars as physical barriers, and secondary metabolites and jasmonate (JA) signaling in chemical defenses. The review discusses genetic and biotechnological approaches to enhance plant resistance, including plant-mediated RNA interference (RNAi) and ectopic expression of insecticidal proteins. These advancements are critical for developing effective and sustainable solutions for whitefly management.

Wang et al. highlight key areas in the study of plant-insect interactions, focusing on the identification of insect elicitors and effectors and their roles in activating plant defense pathways such as jasmonic acid (JA) and salicylic acid (SA) signaling, calcium flux, reactive oxygen species (ROS) bursts, and mitogen-activated protein kinase (MAPK) activation. Multi-omics approaches, including genomics, transcriptomics, and proteomics, are used to analyze insect saliva and salivary glands. The review provides insights into the complex mechanisms of attack and defense and the roles of various elicitors and effectors in these interactions.
